# *De Novo* CD5 Negative Blastic Mantle Cell Lymphoma Presented with Massive Bone Marrow Necrosis without Adenopathy or Organomegaly

**DOI:** 10.1155/2015/146598

**Published:** 2015-08-10

**Authors:** Ghaleb Elyamany, Ali Matar Alzahrani, Eman Al Mussaed, Hassan Aljasem, Sultan Alotaibi, Hatem Elghezal

**Affiliations:** ^1^Department of Central Military Laboratory and Blood Bank, Prince Sultan Military Medical City, P.O. Box 7897, Riyadh 11159, Saudi Arabia; ^2^Department of Hematology and Blood Bank, Theodor Bilharz Research Institute, Giza, Egypt; ^3^Department of Oncology, Prince Sultan Military Medical City, P.O. Box 7897, Riyadh 11159, Saudi Arabia; ^4^Department of Basic Science, Princess Nourah Bint Abdulrahman University, College of Medicine, Riyadh, Saudi Arabia; ^5^Department of Adult Clinical Hematology and Stem Cell Therapy, Prince Sultan Military Medical City, P.O. Box 7897, Riyadh 11159, Saudi Arabia

## Abstract

The recent World Health Organization (WHO) classification defines mantle cell lymphoma (MCL) as a distinct entity characterized by a unique immunophenotype and a molecular hallmark of chromosomal translocation t(11;14)(q13;q32). We report an unusual case of an advanced stage of CD5 negative MCL with a blastoid variant with a massive bone marrow (BM) necrosis as an initial presenting feature, with no adenopathy or hepatosplenomegaly. The pathologic features showed blastoid variant of MCL and flow cytometry showed that the tumor cells were CD5−, CD19+, CD20+, FMC-7+, CD23−, and lambda light chain restricted. Chromosomal analysis, using karyotype and fluorescent in situ hybridization (FISH), demonstrated karyotypic abnormalities in addition to the t(11;14). Our case study may be reported as a unique case of CD5− blastic MCL with unusual presentation and findings which made the diagnosis of MCL difficult.

## 1. Introduction

Mantle cell lymphoma (MCL) is a relatively rare non-Hodgkin's lymphoma (NHL) with a heterogeneous clinical presentation comprising about 3% to 10% of NHL cases [1].

Diagnosis is based on lymph node, bone marrow, or tissue morphology of centrocytic lymphocytes, small cell type, or blastoid variant cells. Chromosomal translocation t(11;14) is the molecular hallmark of MCL; it is considered to be the primary molecular event in juxtaposing the CCND1 gene (11q13) with the IgH gene (14q32) and results in the overexpression of cyclin D1 [2–4]. Although cyclin D1 activation may not be sufficient to induce malignant transformation and additional deregulation of other oncogenes, it is required for lymphoma development. The study of a large series of cytogenetic profiles of MCL revealed that in most cases t(11;14) is associated with numerous structural and numerical alterations of chromosomes considered as secondary rearrangements. The most frequent abnormalities involve chromosomes 1, 7, 11, and 13 [5, 6]. Molecular genetic studies have demonstrated that deletions of 1p21-22, 13q14, and 11q22-q23 are the most common clonal genomic aberrations in the majority of MCL [6, 7].

MCL has characteristics of both indolent and aggressive lymphomas. Because of its aggressive clinical course, MCL identification is important; it has a median survival of 3–5 years [3]. The first line of therapy for MCL is the commonly used regimen of rituximab in combination with chemotherapy and it has a total efficiency of 75%–96% [8, 9].

Here, we describe an unusual case of advanced-stage blastic MCL with a massive BM necrosis as an initial presenting feature with no adenopathy or hepatosplenomegaly (HSM). Chromosomal analysis using karyotype and fluorescent in situ hybridization (FISH) demonstrated complex karyotypic abnormalities in addition to the t(11;14).

## 2. Case Presentation

A 64-year-old male patient, smoker with chronic obstructive airway disease, on bronchodilators, presented with generalized body pain, bilateral lower limb and low back pain, night sweats, anorexia, and weight loss of 25 kg in 6 weeks. He had no fever or chills. By physical examination the patient looked ill and cachectic but had no adenopathy, HSM, or skin lesions.

**Figure 1:**
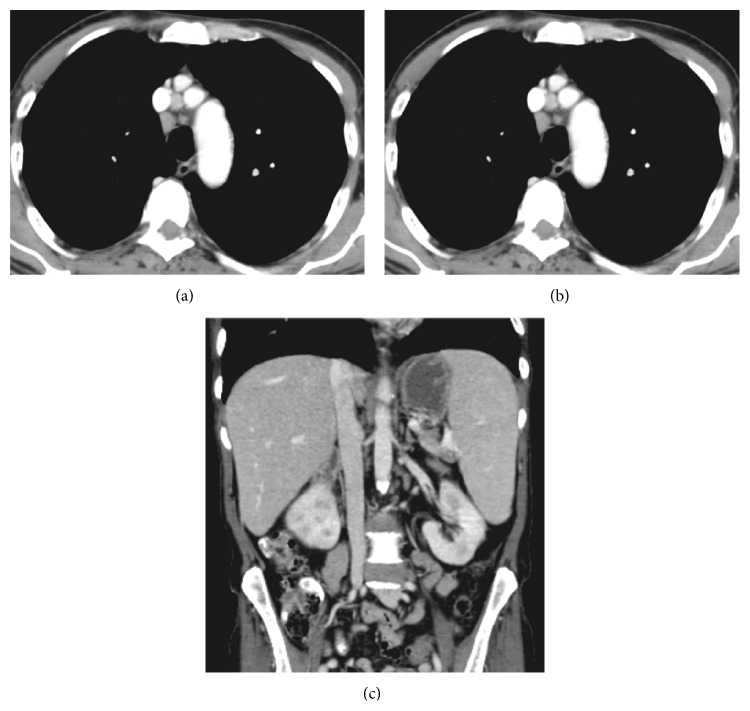
Axial postcontrast CT of the chest ((a) and (b)) shows minimally enlarged mediastinal lymph nodes; the largest one in the right pretracheal region measures about 12.3 × 11.1 mm. Coronal postcontrast CT of the abdomen (c) shows mildly enlarged spleen and liver measuring about 14.5 and 15.7 cm, respectively, without evidence of focal lesions or associated abdominal lymphadenopathy.

A complete blood count revealed anemia (hemoglobin 10.3 gm/dL), thrombocytopenia (90 × 10^9^/L), and mild leukocytosis (12.1 × 10^9^/L) with absolute monocytosis. A peripheral blood film showed no atypical/abnormal lymphocytes, but 1% nucleated RBCs were detected. The white blood cell differential count was neutrophils 7.1, lymphocytes 3.1, monocytes 1.8, basophils 0.1, and eosinophils 0.0. A computerized tomography scan of the chest, abdomen, and pelvis revealed minimally enlarged mediastinal lymph nodes; the largest one in the right pretracheal region measured about 12.3 × 11.1 mm; mildly enlarged spleen and liver measured about 14.5 and 15.7 cm, respectively, without evidence of focal lesions or associated abdominal lymphadenopathy (Figure 1).

**Figure 2:**
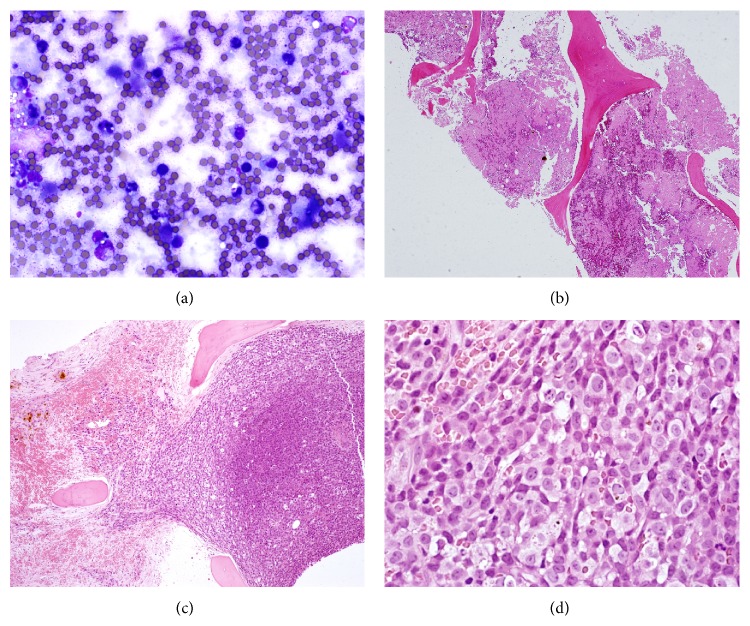
(a) Bone marrow aspirate showing necrotic cells with very rare intact cells. Note intense blue staining of necrotic cells. (b) Bone marrow trephine biopsy showing massive necrosis. Note the homogeneous staining of the necrotic marrow. (c) BM biopsy shows hypercellular marrow due to a diffuse abnormal malignant infiltrate with starry-sky appearance. Note that areas of necrosis are still present. (d) The neoplastic cells show atypical large lymphoid cells with round, slightly irregular, or indented nuclei, dispersed chromatin, abundant agranular cytoplasm, and prominent nucleoli (blast-like cells).

**Figure 3:**
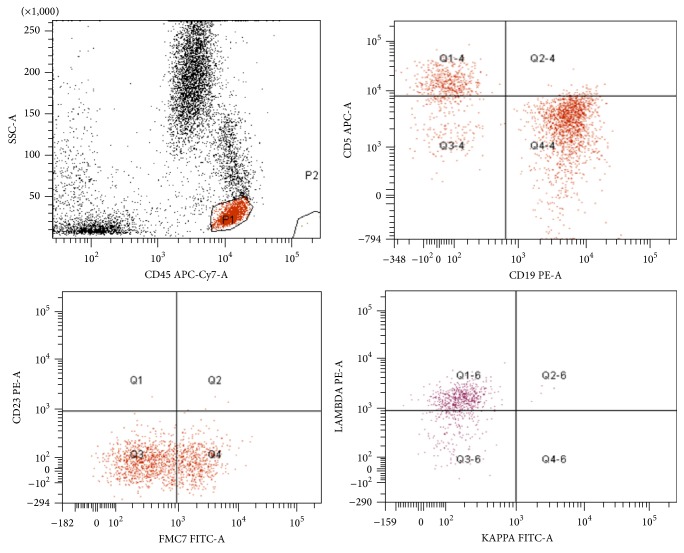
Flow cytometry analysis using side scatter/CD45 gating strategy; the gated population of cells (red) show bright CD45 positivity with expression of CD19 and FMC7 (45%) monotypic lambda light chain. These cells were negative for CD10, CD5, CD23, and CD34.

The first BM smears and biopsy were stained with Wright-Giemsa stain and hematoxylin and eosin and revealed massive BM necrosis (Figures 2(a) and 2(b)). Flow cytometry on peripheral blood (PB) showed no evidence of leukemia or lymphoma involving PB (data not shown). The second BM biopsy revealed hypercellular marrow due to a diffuse abnormal malignant lymphoid infiltrate and areas of necrosis. Morphologically, the neoplastic cells showed atypical large cells with regular or slightly indented nuclei, dispersed chromatin, abundant agranular cytoplasm, and prominent nucleoli (blast-like cells) with starry-sky appearance (Figures 2(c) and 2(d)). Immunophenotyping by flow cytometry revealed a discrete lymphoid population (bright CD45 and low side scatter) that expressed CD19, CD20, CD22, FMC-7 (45%), and monotypic lambda light chain but was negative for CD5, CD10, CD23, and CD34 (Figure 3; some data are not shown). The immunohistochemistry (IHC) was performed (Ventana Medical Systems) and showed positivity for CD45, CD20, cyclin D1, and PAX 5 while CD3, CD5, CD23, bcl-2, and bcl-6 were negative (data not shown).

**Figure 4:**
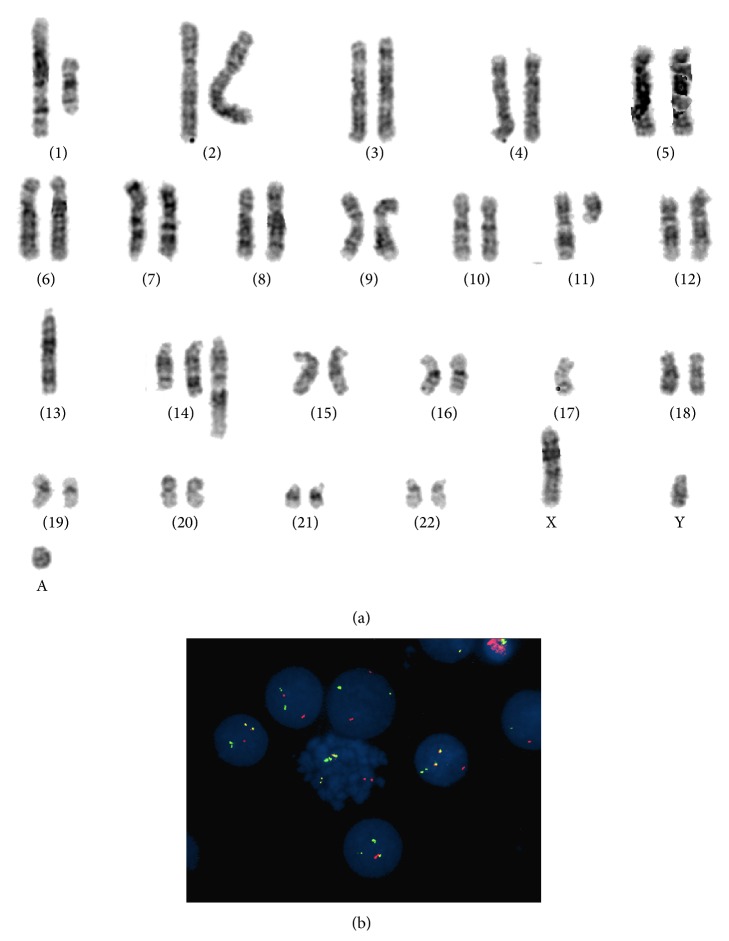
(a) Karyotypic analysis showing complex chromosomal abnormalities including translocation t(11;14). (b) FISH using IGH-CCND dual color dual fusion on interphases and metaphases cells showed the presence of the translocation t(11;14).

In 60% of cells, cytogenetic analysis revealed complex chromosomal abnormalities and chromosomal rearrangements in addition to the t(11;14)(q13;q32) translocation (Figure 4(a)). FISH was performed on interphase cells using Vysis LSI IgH/CCND1 dual color, dual fusion probe (Abbott Laboratories, Abbott Park, IL), and confirmed IgH/CCND1 fusion in 44% of analyzed cells (Figure 4(b)). Cytogenetic investigations were reported as follows: 46,XY,der(1)inv(1)(p32q31)del(1)(p13p32)  [5]/46,XY,sl,t(11;14)(q13;q32)[4]/40,sdl1,-8,der(13)t(13;13)(q12;q34),-13,del(15)(q22),der(16)t(16;?)(p11.2;?),-16,-17,-18,-19[2]/46,XY[9]. nuc ish (CCND1x3), (IGHx3), (CCND1 con IGHx2) [44/100].

The patient received combination chemotherapy CHOP-R (cyclophosphamide, adriamycin, vincristine, prednisone, and rituximab). His condition improved with a long-term survival rate (42 months at the time of publication).

## 3. Discussion

MCL is a clinically aggressive CD5+ B-cell neoplasm. It is highly associated with the t(11;14)(q13;q32) abnormality resulting in deregulated cyclin D1 expression [10, 11].

Although the features of blastic or large cell MCL have each been well described in the literature [12–14], our case of blastic MCL presented with predominant marrow involvement, extensive marrow necrosis without peripheral blood involvement, and without significant adenopathy or HSM. When BM examination represents the initial diagnostic procedure, it requires heightened awareness and the application of ancillary immunologic and molecular techniques. The presence of CCND1 gene rearrangements or cyclin D1 abnormalities should be assessed in such cases to establish the diagnosis of mantle cell lymphoma and exclude other CD5−/CD10− mature B-cell neoplasms.

MCL patients are predominantly male (ratio of 2 : 1 or greater) and have a median age at diagnosis of 60–65 years. Most patients initially present with stages III-IV, which is similar to what was seen in our case [2, 9]; however, our case was unique in its presentation with the absence of lymphadenopathy and extranodal involvement which is usually present in MCL patients [1].

In our case, diagnostic problems were encountered due to massive BM necrosis without viable cells in the first BM examination and unusual clinical presentation with atypical immunophenotypic characteristics and morphologic features in the repeated BM. This prompted initial consideration of acute leukemia, although the immature cell markers CD34, CD10, and TdT were negative. Moreover, there was no access to histology because of no significant lymphadenopathy, organomegaly, or masses. These results are very similar to those of other reported studies [15, 16]. It is recognized that the histologic diagnosis of MCL, particularly in nonnodal cases, may be difficult and that bone marrow histology is not always diagnostically conclusive [17, 18].

It has been shown that approximately 94% of MCL cases carry other karyotypic abnormalities in addition to the t(11;14). In most of these studies, del(13)(q14) was identified as the most frequent abnormality, followed by chromosome 1. Other common abnormalities that have been reported involve chromosomes 3, 6, 9, and 17 [5–7, 19–21].

The t(11;14) translocation is very specific to MCL among other B-NHL and is detected by conventional cytogenetics in 60–75% of MCL cases or by FISH in nearly 100% of MCL cases. Although the presence of the t(11;14) is the hallmark of MCL, it is believed that the resulting overexpression of cyclin D1 is not sufficient to cause a malignant transformation of lymphoid cells on its own. Additional chromosomal aberrations that have been identified in the majority of MCL patients might be the other factors necessary for lymphomagenesis [19, 22, 23], but the significance of these chromosomal abnormalities found in MCL has not been examined extensively [4, 23, 24].

As reported in the literature, CD5− MCL is a diagnostic problem due to its rarity [25]. The ability to distinguish MCL from other B-cell lymphomas is extremely important because of the differences in the treatment options and in the clinical course [26]. Cytogenetic studies confirm the presence of t(11;14) with a complex karyotype. These findings highlight the importance of using ancillary techniques such as molecular and cytogenetic studies in such cases to establish the diagnosis.

## 4. Conclusions

Our case study may be reported as a unique case of CD5 negative blastic MCL with massive bone marrow necrosis as an initial feature without significant lymphadenopathy or HSM. These unusual findings made the diagnosis of MCL difficult. This study also highlights the importance of using ancillary techniques such as flow cytometry and molecular and cytogenetic studies to establish the diagnosis and differentiate different types of lymphomas in such cases. Most studies of MCL report karyotypic abnormalities in addition to the t(11;14), but the significance of these chromosomal abnormalities found in MCL has not been examined extensively and they might be factors necessary for lymphomagenesis.

## Abbreviations

WHO: The World Health Organization
MCL: Mantle cell lymphoma
NHL: Non-Hodgkin's lymphoma
HSM: Hepatosplenomegaly
FISH: Fluorescent in situ hybridization
CHOP-R: Cyclophosphamide, adriamycin, vincristine, prednisone, and rituximab
PB: Peripheral blood
BM: Bone marrow
IHC: Immunohistochemistry.
